# Abnormalities in perineuronal nets and behavior in mice lacking CSGalNAcT1, a key enzyme in chondroitin sulfate synthesis

**DOI:** 10.1186/s13041-017-0328-5

**Published:** 2017-10-05

**Authors:** Nozomu Yoshioka, Shinji Miyata, Atsushi Tamada, Yumi Watanabe, Asami Kawasaki, Hiroshi Kitagawa, Keizo Takao, Tsuyoshi Miyakawa, Kosei Takeuchi, Michihiro Igarashi

**Affiliations:** 1 Department of Neurochemistry and Molecular Cell Biology, Niigata University Graduate School of Medical and Dental Sciences, 1-757 Asahimachi, Chuo-ku, Niigata, 951-8510 Japan; 2 Transdiciplinary Research Program, Niigata University, Asahi-machi, Niigata, 951-8510 Japan; 3 Department of Biochemistry, Kobe Pharmaceutical University, Motoyamakita-machi, Kobe, 658-8558 Japan; 4 Institute for Advanced Research, Nagoya University, Furo-cho, Nagoya, 464-8601 Japan; 5 PRESTO, Japan Science and Technology Agency (JST), Chiyoda-ku, Tokyo, 102-0075 Japan; 6 Section of Behavior Patterns, National Institute of Physiological Sciences, Okazaki, Aichi 444-8787 Japan; 7 Division of Experimental Animal Resource and Development, Life Science Research Center, Toyama University, Toyama, 930-0194 Japan; 8 Division of Systems Medical Science, Institute for Comprehensive Medical Science, Fujita Health University, Toyoake, Aichi 470-1192 Japan; 9 Department of Medical Biology, School of Medicine, Aichi Medical University, Nagakute, Aichi 480-1103 Japan; 10 Present address: Divisions of Neurobiology and Anatomy, Niigata University Graduate School of Medical and Dental Sciences, Niigata, Japan; 11 Present address: Divisions of Preventive Medicine, Niigata University Graduate School of Medical and Dental Sciences, Niigata, Japan

## Abstract

Chondroitin sulfate (CS) is an important glycosaminoglycan and is mainly found in the extracellular matrix as CS proteoglycans. In the brain, CS proteoglycans are highly concentrated in perineuronal nets (PNNs), which surround synapses and modulate their functions. To investigate the importance of CS, we produced and precisely examined mice that were deficient in the CS synthesizing enzyme, CSGalNAcT1 (T1KO). Biochemical analysis of T1KO revealed that loss of this enzyme reduced the amount of CS by approximately 50% in various brain regions. The amount of CS in PNNs was also diminished in T1KO compared to wild-type mice, although the amount of a major CS proteoglycan core protein, aggrecan, was not changed. In T1KO, we observed abnormalities in several behavioral tests, including the open-field test, acoustic startle response, and social preference. These results suggest that T1 is important for plasticity, probably due to regulation of CS-dependent PNNs, and that T1KO is a good model for investigation of PNNs.

**Electronic supplementary material:**

The online version of this article (10.1186/s13041-017-0328-5) contains supplementary material, which is available to authorized users.

## Introduction

Chondroitin sulfate (CS) is one of the most abundant glycosaminoglycans, which are composed of long, repeated disaccharide chains. For synthesis of CS proteoglycans (CSPGs), CS is attached to core proteins, such as aggrecan (AGR), neurocan, phosphacan, and versican. These CSPGs are mainly localized in the extracellular matrix. In the central nervous system, CSPG-enriched areas are called perineuronal nets (PNNs), which are specialized structures that surround synapses and are specifically recognized by *Wisteria floribunda* lectin (WFA). PNNs modulate synaptic functions, particularly GABAergic inhibitory input via parvalbumin (PV) (+) neuronal cells [1]. Recently, the function of PNNs in the regulation of synaptic plasticity and memory [2, 3] has been investigated, and PNN abnormalities in relation to human mental diseases have been reported [4–6].

CS synthesis is sequentially performed by approximately 15 enzymes including glycosyltransferases and sulfotransferases in three steps: (I) tetrasaccharide linker synthesis attached to the core protein; (II) disaccharide repeat synthesis for GalNAc (*N*-acetylgalactosamine)-GlcUA (glucuronic acid); and (III) sulfation of these sugars. The first step is shared with another glycosaminoglycan, heparan sulfate; thus, the first unique step in CS synthesis is GalNAc transfer to the linker. CSGalNAcT1 (T1) is a key enzyme in CS synthesis, because this enzyme catalyzes the first unique step in CS synthesis, apart from heparan sulfate synthesis.

We previously produced T1 knockout mice (T1KO; [CSGalNAcT1^−/−^] mice) and examined some functions of CS [7]. T1KO mice have impaired bone development and a 10% smaller body size than wild-type (WT) mice [7, 8]. They have the striking feature of rapid recovery following spinal cord injury [9]. During recovery from spinal cord injury in T1KO, we observed a loss of PNNs in the spinal cord [9], suggesting abnormal PNN formation.

Here, we systematically examined the T1KO brain. Biochemically, the amount of CS was reduced by about half in each region in T1KO brain. Histologically, abnormal PNNs due to reduced CS were observed, and the mice demonstrated abnormal behaviors in several tests. Taken together, we conclude that T1 plays an important role in supplying CS for PNN development and brain functions related to several characteristic behaviors.

## Results

### Biochemical and histochemical characteristics of T1KO

**Fig. 1:**
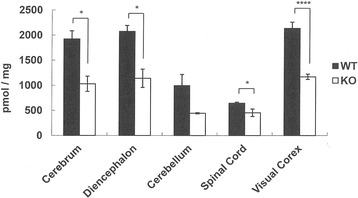
T1KO had half the amount of CS compared to WT in various brain regions. The total amount of CS in WT (*closed bars*) and in T1KO (*open bars*) is shown. The mice used were 12 week-old males. Data are the mean ± SEM. *p < 0.05; ***p < 0.001; *n.s.*, not significant. *N* = 3–4 mice each

T1KO mice were fertile and showed no macroscopic abnormalities in the brain. However, various regions of the brain had only 50% of the amount of CS compared to WT, including significant differences in the cortex and diencephalon (Fig. 1).

Disaccharide compositions were also analyzed, and among five disaccharide patterns of CS (ref. 16; i.e., CS-O, −A, −C, −D, and -E) [7, 8, 10], we observed a slight but significant increase in the CS-E ratio in both the cortex and diencephalon of the T1KO (Table 1). These results suggest that T1KO is an important enzyme for CS synthesis in the brain. Microscopic abnormalities in T1KO brain.

**Table 1:** The composition of CS disaccharides in T1KO compared with WT

mol%	Average	SEM	Average	SEM	*P* value
Cerebrum	WT		KO		
O	9.1	0.19	12.0	1.04	*0.046*
C	1.8	0.27	5.3	0.12	0.821
A	87.2	0.14	77.7	0.69	*0.027*
D	0.7	0.05	2.1	0.03	*0.003*
E	1.2	0.05	2.9	0.44	*0.001*
Total	100.0		100.0		
Diencephalon	WT		KO		
O	4.4	0.17	4.7	0.25	0.374
C	3.8	0.09	3.0	0.06	*0.002*
A	88.9	0.34	89.4	0.34	0.367
D	1.3	0.13	1.0	0.03	0.075
E	1.6	0.05	1.9	0.06	*0.030*
Total	100.0		100.0		
Cerebellum	WT		KO		
O	7.6	1.88	4.5	0.24	0.183
C	3.7	0.17	4.9	0.43	0.062
A	85.5	1.52	87.3	0.46	0.333
D	1.8	0.20	1.6	0.12	0.395
E	1.4	0.01	1.7	0.15	0.082
Total	100.0		100.0		
Spinal Cord	WT		KO		
O	12.0	1.04	11.8	2.68	0.930
C	5.3	0.12	4.7	0.06	*0.041*
A	77.7	0.69	78.7	2.27	0.647
D	2.1	0.03	1.4	0.25	*0.036*
E	2.9	0.44	3.4	0.59	0.525
Total	100.0		100.0		
Visual Cortex	WT		KO		
O	9.0	0.18	10.7	0.16	*4E-04*
C	2.7	0.01	2.3	0.03	*3E-05*
A	86.4	0.18	84.6	0.19	*5E-04*
D	0.7	0.03	0.4	0.03	*4E-04*
E	1.2	0.01	1.9	0.03	*5E-07*
Total	100.0		100.0		

Note that among the five patterns of CS-derived disaccharides, CS-E in T1KO was significantly increased in each brain region. Statistically significant differences in T1KO are shown in italics (*p* < 0.05), compared to the corresponding results for WT [7, 8, 10]

**Fig. 2:**
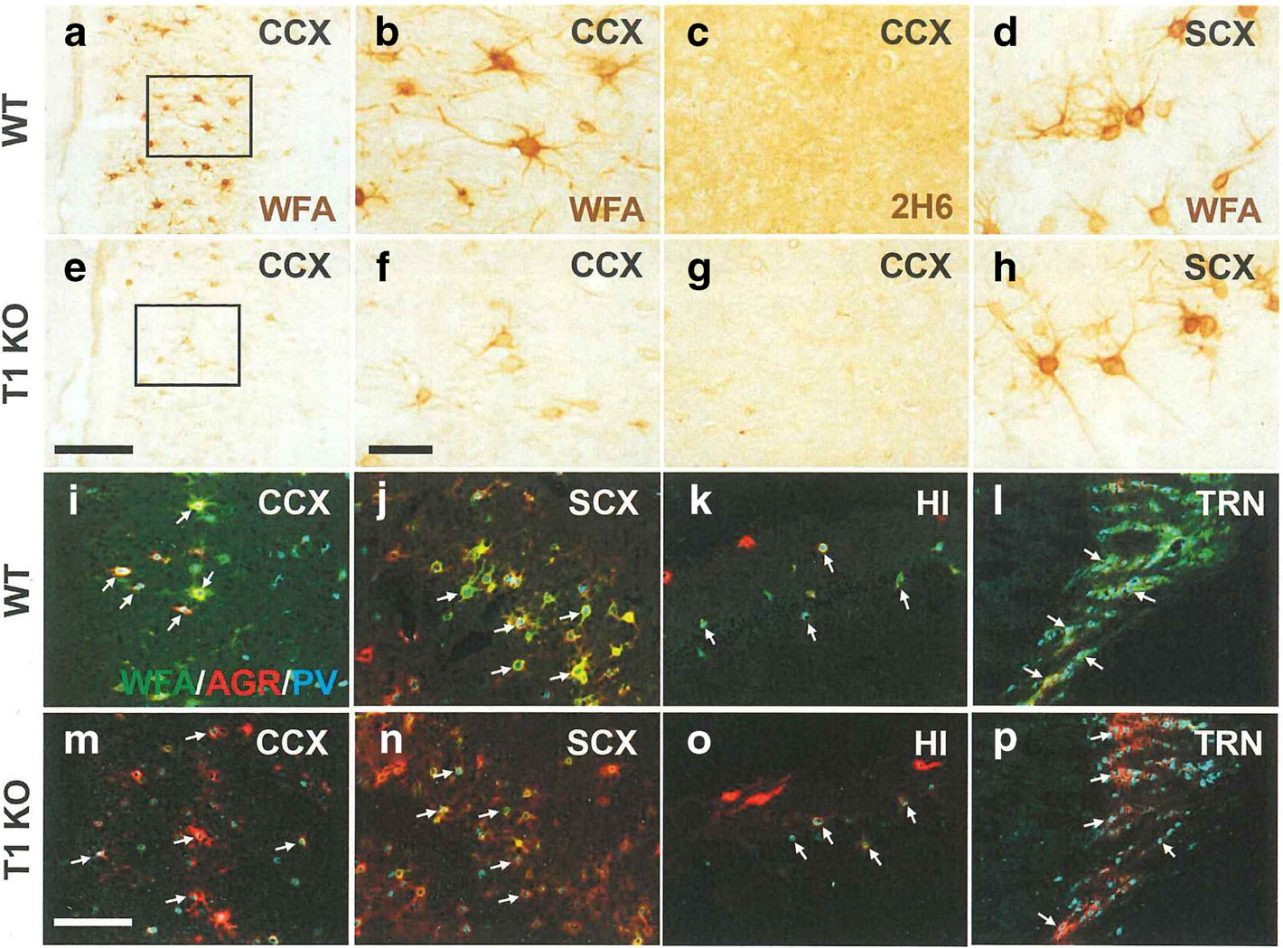
Representative views of histochemistry revealed abnormal PNNs but normal PV cells and AGR distribution in various brain regions. **a-p** DAB staining of CS in the cingulate cortex (CCX; WT; **a-c,** T1KO; **e-g**) and in the somatosensory cortex (SCX; WT: **d**, T1KO: **h**). **b** and **f** are higher magnification views of the rectangular areas in (**a)** and (**e**)**,** respectively. CS was labeled with WFA (**a, b, d, e, f, h**), and directly recognized with an anti-CS antibody, 2H6 (**c, g**). **i-p** Triple fluorescent staining with WFA (*green*), anti-AGR (*red*), and anti-PV (*blue*) in the CCX (**i, m**), SCX (**j, n**), hippocampus (HI) (**k, o**), and thalamic reticular nucleus (TRN) (**l, p**) in the brain obtained from WT (**i-l**) and T1KO (**m-p**) mice. Representative PV cells were indicated by *white arrows* (**i-p**). Scale bars, 200 μm (**a** and **e**), 50 μm (**b-d, f-h**), and 100 μm (**i-p**)

Next, we examined whether microscopic abnormalities were found in T1KO brain. Because high levels of CS should be detected in PNNs of WT, we histochemically examined WFA-labeled PNNs using diaminobenzidine (DAB) staining and fluorescent studies. DAB staining of WFA, which represents PNNs, showed that PNNs were largely reduced in the cingulate cortex of T1KO (Fig. 2a, b, e-f), and to some extent in the somatosensory cortex of T1KO (Fig. 2d and h). Using 2H6, an antibody against CS that also tends to label extracellular matrix other than PNNs, we confirmed that CS staining in such areas was much reduced (Fig. 2c and g).

We quantitatively analyzed WFA, anti-AGR, and anti-PV immunofluorescence by assessing the intensity (Fig. 2i-p) following triple fluorescent staining. Somatosensory cortex showed a reduction in WFA (+) PNNs (Fig. 2i and m), but the intensities of anti-AGR and anti-PV immunofluorescence, which is thought to represent the amounts of AGR in PNNs and PV (+) cells, respectively, were not different in T1KO (Fig. 2m). We observed similar patterns in the cingulate cortex (Fig. 2j and n), hippocampus (Fig. 2k and o), and thalamic reticular nucleus of the T1KO (Fig. 2l and p). These results suggest that T1KO have reduced CS in PNNs but the core proteins remain, independent of CS.

**Fig. 3:**
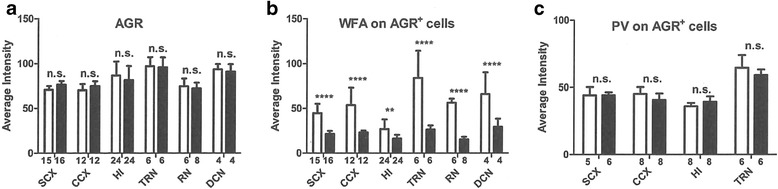
Quantitative analysis of the histochemistry. **a-c** Intensity analyses of AGR (**a**), WFA (**b**), and PV (**c**) relative to AGR (+) pixels. The average intensity on each section was calculated. *Open bars*, WT; *Closed bars*, T1KO. The data represent the mean ± SD. RN, red nucleus; DCN, deep cerebellar nucleus. See Fig. 2 for other abbreviations. The number of samples for each data point is shown at the bottom of the column. **p < 0.01; ****p < 0.0001; *n.s.*, not significant. Bonferroni post-hoc tests after two-way factorial ANOVA

Quantitative analysis of these fluorescent results (Fig. 2i-p) confirmed this observation (Fig. 3). In all examined areas in T1KO, the intensities of anti-AGR and anti-PV were not changed, but the intensity ratios of WFA (+) PNNs per AGR (+) area were selectively decreased (Fig. 3). We also confirmed that AGR immunoreactivity was not changed between brains of WT and T1KO using immunoblotting (Additional file 1 Figure S1).

### Behavioral abnormalities in T1KO

**Fig. 4:**
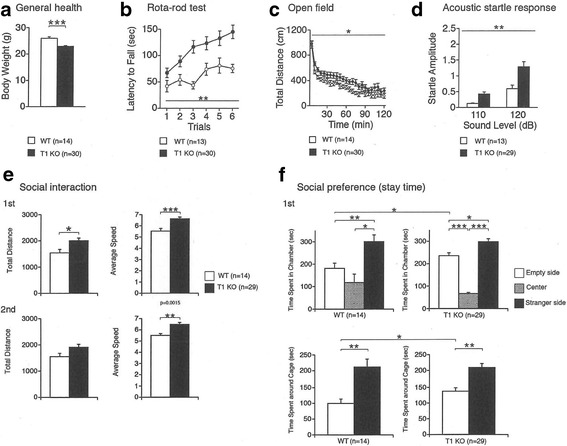
Behavioral abnormalities in T1KO. Significant differences were observed between genotypes in the six behavioral tests shown here. **a** General health (body weight; ****p < 0.0001), **b** Open field test (**p < 0.05), (**c**) Rota-rod test (**p < 0.05), **d** Acoustic startle responses (**p < 0.05), **e** General activity (**p < 0.05; ***p < 0.001), and **f** Social preference (stay time) (**p < 0.05; ***p < 0.001; ****p < 0.0001). Each group was composed of 20 male mice at the age of 12 weeks. See also Table 2

We performed a battery of more than 20 behavioral tests and found the following abnormalities in T1KO compared to WT: general health (body weight), open field test, rota-rod test, acoustic startle responses, general activity, and social preference (stay time) (Fig. 4). The total distance traveled in the open field test, which measures voluntary activity in a novel environment, was significantly higher in T1KO than WT, suggesting that T1KO showed hyperlocomotive activity. Acoustic startle tests, which measure reflex movement when a sudden loud sound stimulus is given, revealed that T1KO had much larger responses than WT. In contrast, other tests including learning and memory were not impaired (Table 2). These results suggest that T1KO show several abnormal behaviors that involve higher brain functions, probably due to the reduced CS concentration in PNNs.

**Table 2:** The behavioral tests in T1KO that were not significantly different between genotypes (*p* ≥ 0.05)

Test	Controls		Mutants		F value	*p* value
	Mean	SEM	Mean	SEM		
General health/neurological screen						
body temperature (°C)	36.85	0.141	37.28	0.164	2.747	0.1049
grip strength (n)	0.554	0.02	0.577	0.012	1.038	0.3141
wire hang (latency to fall, s)	39.512	5.428	29.507	2.879	3.204	0.0808
Light/dark transition						
latency to light (s)	44.143	5.829	56.1	6.621	1.293	0.2619
light distance (cm)	622.129	46.667	609.563	41.119	0.034	0.8548
dark distance (cm)	1555.886	65.493	1628.983	35.355	1.147	0.2904
number of transitions	32.714	2.23	28.967	1.965	1.32	0.2571
stay time in light (s)	158.071	9.869	147.5	10.065	0.423	0.5189
Open field						
stereotypic counts	6911.643	520.353	7369.367	526.983	0.289	0.5939
vertical activity	348.357	53.683	275.4	43.994	0.966	0.3312
center time (s)	633.986	121.934	813.077	111.923	0.945	0.3365
Elevated plus maze						
number of total entry (times)	36.071	2.319	36.833	2.229	0.044	0.835
entry into open arms (%)	13.393	2.567	13.863	1.572	0.027	0.8715
total distance travelled (cm)	1806.893	92.545	1880.087	73.559	0.343	0.5614
time on open arms (times)	3.443	1.125	2.923	0.744	0.152	0.6985
Hot plate						
latency (s)	6.403	0.386	6.318	0.394	0.018	0.8944
Social interaction (novel environment)						
duration of contacts (s)	68.7	9.138	88.707	7.29	2.605	0.1222
number or contacts (times)	40.143	3.845	45.333	2.462	1.358	0.2577
mean duration of contacts (s)	1.786	0.263	1.993	0.187	0.4	0.5341
distance travelled (cm)	2957.586	285.871	3196.793	153.133	0.654	0.4283
Porsolt forced swim						
day1 immobility (%)	41.8131	3.617	46.252	2.314	1.099	0.3009
day2 immobility (%)	57.747	3.627	54.268	2.624	0.569	0.4551
Gait analysis						
front swing/stride duration	41	0.508	40.969	0.43	0.002	0.9662
front brake/stride duration	28.115	0.909	27.234	0.715	0.512	0.4784
front propel/stride duration	30.896	0.871	31.795	0.645	0.635	0.4302
front stance width	1.946	0.062	1.921	0.038	0.134	0.7164
front step angle	62.308	1.762	60.066	1.075	1.269	0.2666
front paw angle	−0.235	0.866	0.89	0.455	1.591	0.2144
hind brake/stride duration	14.142	0.831	15.564	0.364	3.373	0.0737
hind step angle	53.746	1.757	55.321	1.627	0.338	0.564
hind paw angle	−0.788	0.953	−0.983	0.519	0.038	0.8471
Barnes maze						
time spent around target (probe test, 1 day)	40.538	4.748	39.143	4.067	0.042	0.8385
time spent around target (probe test, 1 month)	32.154	5.78	30.172	4.224	0.072	0.7904
time spent around target (reversal test, 1 day)	31.077	4.118	28.214	3.227	0.27	0.6064
Cued and contextual fear conditioning						
conditioning (freezing, %)	57.756	2.66	56.821	1.83	0.82	0.7764
context test, 1 day (freezing, %)	70.082	3.444	66.333	3.372	0.453	0.5053
cued test, with tone,1 day (freezing, %)	89.158	3.641	88.147	2.004	0.069	0.7945
context test, 30 days (freezing, %)	79.412	5.225	79.481	3.222	0.0001376	0.9907
cued test, pretone period, 30 days (freezing, %)	60.603	4.475	49.672	4.306	2.345	0.1342
cued test, with tone, 30 days (freezing, %)	90.183	3.079	87.86	1.944	0.425	0.5186
Tail suspension						
immobility (%)	37.255	3.747	41.75	2.603	0.957	0.334
24 h home cage monitoring						
activity light period (A.U.)	478,882.065	30,477.038	670,800.611	107,540.908	2.948	0.1167
activity dark period (A.U.)	1,033,998.833	66,355.759	1,385,925	191,066.128	3.027	0.1125
number of particle dark period	1.175	0.02	1.196	0.022	0.482	0.5034
	Stranger		Empty or Familiar		t value	*p* value (paired-t)
	Mean	SEM	Mean	SEM		
Social approach test (Stranger vs Empty)						
number of entry around cage (controls)	9	1.043	7.714	0.952	1.178	0.26
time spent around cage (mutants)	209.241	12.352	137.448	10.928	0.627	0.5359
Social novelty preference test (Stranger vs Familiar)						
number of entry around cage (controls)	8.357	0.887	8.786	1.11	−0.405	0.6918
number of entry around cage (mutants)	9.862	0.851	10.034	0.863	−0.16	0.8742
time spent around cage (controls)	165.071	16.433	148.5	28.882	0.435	0.6708
time spent around cage (mutants)	175.31	17.16	156.379	13.814	0.673	0.5063

The tests that were significantly different between T1KO and WT are displayed in Fig. 4. The results of the tests that were not significantly different are listed here. Each group was composed of 20 male mice at the age of 12 weeks

## Discussion

Here, we showed both biochemical (Fig. 1; Table 1) and histochemical results (Figs. 2 and 3) demonstrating reduced CS in various areas of the T1KO brain. Histochemical results also revealed that the CS reduction was in PNNs of the various T1KO regions in brain, in the absence of a change in the amount of AGR (Figs. 2 and 3). In addition, several types of mouse behaviors were significantly abnormal in T1KO (Fig. 4), suggesting that these tests may reflect CS- and PNN-dependent changes. Regarding the CSPG species, we should consider membrane-bound proteins, such as receptor protein phosphatases [10]. However, the total amount of CSPG species is likely considerably less than those of PNNs, considering the large space occupied by the extracellular matrix. Thus, our demonstration that T1KO have reduced PNNs is important.

Reductions in CS by at least 50% were observed in various T1KO brain regions, suggesting that T1 is an important enzyme responsible for CS synthesis (Fig. 1; Table 1), although five other enzymes with similar substrate specificities were reported [10]. As far as we know, this is the first example of genetically targeted mice that showed a 50% loss in the total amount of CS. These results support the hypothesis that T1 is the most important enzyme for CS synthesis in the brain.

In addition, the CS-E ratios slightly but significantly increased in cerebrum and diencephalon of T1KO (Table 1). The exact reason of this change is not clearly known, however, it is likely that loss of T1 affects the following sulfation processes by sulfatases [10]. In PNNs, CS-E is reported to be involved in association of semaphorin-3A [11], not only quantitative decrease but also such qualitative changes of PNNs may have some effects on the behavioral abnormalities (Fig. 4).

We also showed that T1KO generally had reduced WFA (+) PNNs in various brain areas, although the decrease was somewhat variable (Figs. 2 and 3). Thus, T1-dependent synthesis of CS is more prevalent in PNNs. In such cases, we did not detected abnormalities of the typical CSPG core protein, AGR, or another glycosaminoglycan, hyaluronic. These results suggest selective reduction of CS chains in PNNs in T1KO.

T1 enzyme deficiency caused behavioral abnormalities including abnormalities in the open field test, social interaction test, and acoustic startle response (Fig. 4). These results suggest that CS reduction in PNNs by T1 loss caused abnormalities in higher brain functions and hyperlocomotive activity in mice. These effects are thought to be due to an increase in excitability induced by impaired GABAergic functions [12]. In addition, Ito et al. reported that chronic stress inhibits GABAergic signaling [12]. Thus, although the exact relationship between each behavioral abnormality and reduced CS in PNNs is not fully understood, one possibility is that the balance in excitatory (glutamatergic) and inhibitory (GABAergic) inputs is shifted in abnormal PNNs, because in various regions, the functions of PV(+) cells which are known to be GABAergic interneurons, are regulated by PNN activity [13].

It is also recently reported that decreased GABAergic functions induce the sociability [14], which may be involved in the changes of our results concerning social interaction and preference (Fig. 4e-f), as models of human autism-spectrum and schizophrenia [15–17].

In our adult T1KO, fear conditioning memories were not significantly impaired (Fig. 4), in contrast to a previous report in which loss of CS in PNNs by chondroitin’s ABC (ChABC) erased these memories in mice younger than 3 weeks [18]. Such a “critical period” in mice may have different mechanisms in adult vs. juvenile mice [19]. Using these same T1KO, we recently showed that ocular dominance plasticity in the critical period was severely impaired after monocular deprivation, probably due to reduced function of PV (+) cells in the visual cortex [20]. In mice aged 3 weeks for these experiments, AGR and PV (+) cells were reduced, together with CS in PNNs in the visual cortex [20], which is different from our current results in adult mice (Figs. 2 and 3). As shown in Figs. 2 and 3, some of these morphological abnormalities were observed and some were not. Taken together, T1KO may have additional behavioral abnormalities in the developmental stage, and may be a good model for studies of PNN-dependent neural plasticity. A report that the good results of open filed tests as higher brain functions, after learning, are reported to induce the increase of PNNs [21], seems related to our results (Fig. 4c).

Consistent with the above mention of chronic stress [12], PNNs may be also involved in human psychiatric disorders [22], and this idea should be confirmed using an appropriate animal model for PNNs. However, until now, only the ChABC-treated mouse model represented decreased PNNs. The problem with this acute model for inducing PNN abnormalities is that the extent of the decreases in CS in PNNs and how long this effect is maintained are unknown. In contrast, our T1KO mice quantitatively exhibited a 50% reduction in CS (Fig. 1) and a decrease in CS in PNNs, but the absence of a decrease in AGR (+) area or PV (+) cells (Figs. 2 and 3). Thus, T1KO may be a better model for abnormal PNNs than ChABC-treated mice. Recently, in the KO of a link protein Bral2, a component of PNNs, abnormal acoustic startle tests are reported [23], may support our results (Fig. 4d).

Taken together, we conclude that T1 is the most important factor that controls CS-dependent brain functions. In addition, T1KO may be the most suitable genetic model for studying the functions of PNNs. To further examine the mechanism of CS synthesis and its biological significance, we are planning to analyze mice that are null for CSGalNAcT2 (T2), which is the other isoform of T1, and/or conditional T1/T2 KO mice. For example, most learning and memory tests in T1KO did not show a significant difference from WT, probably because they are dependent upon hippocampal activity [15]. In T1KO, hippocampal PNNs were significantly but only slightly reduced compared to PNNs in other brain regions (Figs. 2 and 3). T1/T2 double knockouts will probably be embryonic lethal due to induction of skeletal defects (M.I., in preparation). However, using T2KO, T1KO/T2 heterozygotes, and T1 heterozygotes/T2KO, larger effects of decreased CS in PNNs are expected.

## Methods

### Animals

T1KO, T1 heterozygotes, and WT littermates on the C57BL/6 background were maintained [7, 9]. Four T1KO or four WT mice were housed per cage, and the mice were group-housed in a room with a 12-h light/dark cycle.

### Quantitative biochemical analysis of CS

Purification and quantification of CS in various areas of the brain were performed as described previously [9]. In brief, the mice were sacrificed by decapitation. Various regions of the adult mouse brain were dissected, homogenized, and treated with protease (0.01 mg actinase E, 10 mM CaCl_2_, 50 mM Tris-HCl, pH 8.0) for 2 days at 55 °C. After trichloroacetic acid treatment, the supernatant was gel filtered (Sephadex G-25, 8.3 ml (PD-10 column), and the flow-through fractions were collected. To analyze the disaccharide composition, fractions were treated with ChABC (5 mIU ChABC in 60 mM CH_3_COONa, 50 mM Tris-HCl, pH 8.0) for 12 h at 37 °C. The disaccharides were labeled with 2-aminobenzamide (350 mM 2-aminobenzamide, 1 M NaCNBH_3_ in DMSO/acetic acid (7:3)) for 2 h at 65 °C and analyzed quantitatively using high-performance liquid chromatography (column: YMC pack PA, 4.6 × 250 mm, elution: 16–530 mM NaH_2_PO_4_, flow rate: 1 ml/min; detection: 330 nm excitation and 420 nm emission). The disaccharide analysis data are shown in Table 1.

### Histochemistry and immunohistochemistry

Histological procedures were carried out according to previously described methods [24]. Antibodies against AGR (rabbit polyclonal) and PV (mouse monoclonal) were purchased from Millipore, and from Swant Inc. (Switzerland), respectively. For tissue preparation, under deep anesthesia with isoflurane, the brain was fixed by cardiac perfusion with PBS followed by ice-cold 4% paraformaldehyde in 0.1 M phosphate buffer, pH 7.4. The brain was dissected out, immersed in the same fixative overnight, and transferred to 20% sucrose in 20 mM PBS, pH 7.4 until it sank. Each brain was frozen in crushed dry ice, and 30 μm-thick consecutive coronal sections were cut on a cryostat and stored at −20 °C until histological staining.

Sections were initially rinsed in 20 mM PBS and incubated in 0.1% Triton X-100 for 15 min at room temperature. After rinsing in 20 mM PBS, the sections were incubated overnight at 4 °C with a mixture of biotinylated-WFA (Sigma-Aldrich) and primary antibodies diluted with 20 mM PBS containing 0.5% skim milk. After rinsing in 20 mM PBS for 15 min, sections were incubated with a mixture of streptavidin-Alexa Fluor® 647 conjugate (1:100; Molecular Probes, Inc., Eugene, OR) and secondary antibodies conjugated to Alexa Fluor®488 or Alexa Fluor®594 (1:100; Jackson ImmunoResearch Laboratories, West Grove, PA) for 60 min at 37 °C. After rinsing with 20 mM PBS for 15 min, sections were mounted on MAS®-coated glass slides (Matsunami Glass, Osaka, Japan) and coverslipped with ProLong Gold (Molecular Probes). Sections were observed and digital images were recorded on a confocal laser scanning microscope (FV-1200, Olympus, Tokyo, Japan). TIF files were processed with Photoshop software (Adobe, San Jose, CA). Both brightness and background were adequately adjusted.

For DAB staining, free-floating sections were initially rinsed in 20 mM PBS and incubated in a mixture of 3% hydrogen peroxide and 0.1% Triton X-100 for 15 min at room temperature. 2H6 (Seikagaku Corporation, Tokyo, Japan) was diluted in 20 mM PBS containing 0.5% skim milk. For 2H6 staining, following rinsing in 20 mM PBS for 15 min, sections were incubated with biotinylated anti-mouse IgM secondary antibody (1:200; Vector Laboratories, Burlingame, CA) for 30 min at 37 °C. After rinsing with 20 mM PBS for 15 min, sections were incubated in avidin-biotin peroxidase complex (Vectastain ABC kit, Vector Laboratories) for 30 min (2H6) or 60 min (WFA) at 37 °C. After rinsing with 20 mM PBS, immunoreaction was visualized in 50 mM Tris-HCl buffer (pH 7.4) containing 0.01% DAB and 0.01% hydrogen peroxide at 37 °C for 5–10 min. Sections were mounted on MAS-coated glass slides (Matsunami Glass), air-dried on a hot plate at 40 °C, and coverslipped with Entellan Neu (Merck, Darmstadt, Germany) after dehydration through ethanol and xylene.

Dilution ratios: were as follows: WFA, 1:50; 2H6, 1:100; anti-PV, 1:500; and anti-AGR, 1:500.

### Quantitative morphometry

We initially counted the number of PV (+) cells. However, that method was not suitable for exact quantification of PV (+) cells because the staining of PV (+) cells varied widely. Thus, we switched to the following quantification methods. The fluorescent intensity of the immunohistochemical and WFA histochemical images was statistically calculated using Image J. Double- (AGR and WFA) or triple (AGR, WFA, and PV)-labeled confocal images were used for the intensity analysis. AGR+ pixels were extracted by masking with an intensity threshold (>50). The average intensity of the AGR+ pixels was calculated for each AGR, WFA, or PV image. The average intensity data were grouped by genotype and brain area. Two-way factorial ANOVA with Bonferroni post-hoc tests were performed for statistical analysis.

### Animal behavioral tests

A battery of mouse behavioral tests was done as described previously [25, 26]. All behavioral tests were carried out with male mice. A general health check and neurological screen were conducted as previously described [27]. The order in which mice were subjected to tests was counterbalanced. Raw data were disclosed in the Mouse Phenotype Database (http://www.mouse-phenotype.org/). T1KO and WT mice (*n* = 20 each) at 8 weeks of age were tested. The tests that showed statistically significant differences in T1KO vs. WT were:

(I) Open field test: This test was performed to evaluate locomotor activity and emotional response [21]. The apparatus was a transparent square cage (42 × 42 × 30 cm; Accuscan Instruments, Columbus, OH). The center of the floor was illuminated at 100 lx. Each mouse was placed in the open field apparatus and recorded for 120 min. Total distance traveled (cm), vertical activity (rearing measured by counting the number of photobeam interruptions), time spent in the center area (20 × 20 cm), and the beam-break counts for stereotyped behaviors were measured.

(II) Social interaction test in a novel environment [27]: Two mice of the same genotype that were previously housed in different cages were placed in a box together (40 × 40 × 30 cm; O’Hara & Co., Tokyo, Japan) and allowed to explore freely for 10 min. Mouse behavior was analyzed automatically using ImageSI software. The total duration of contacts (s), number of contacts, total duration of active contacts (s), mean duration per contact (s), and total distance traveled (cm) were measured. Active contact was defined as follows: images were captured at three frames per second, and distance traveled between two successive frames was calculated for each mouse. If the two mice contacted each other and the distance traveled by either mouse was 5 cm or more, the behavior was considered an “active contact.”

(III) Three-chambered social approach test: The test for sociability and social novelty preference was conducted as previously described [28, 29]. For habituation to the test environment, stranger mice were placed in a small cylindrical cage with vertical bars that was put in a corner of the chamber prior to the test. Test mice were placed in the middle chamber and allowed to explore and habituate to the chambers for 10 min just before the first session. In the first session, an unfamiliar mouse (stranger 1) was put in the cage and placed in a corner of the left or right side chamber. The selection of the left or right side chamber was counterbalanced across test mice. An empty cage was placed in the corner of the other chamber. Then the test mouse was placed in the middle chamber and allowed to explore the three chambers for 10 min. In the second session, another unfamiliar mouse (stranger 2) was placed in the cage that had been empty during the first session. Then the test mouse was placed in the middle chamber and allowed to explore for 10 min. Total distance travelled, average locomotion speed, and the amount of time spent around the cages were measured. Data acquisition and analysis were performed automatically using ImageSI.

(IV) Startle response/prepulse inhibition test: A startle reflex measurement system (O’Hara & Co.) was used to measure startle response to a loud noise and prepulse inhibition of the startle response. A test session began by placing a mouse in a plastic cylinder where it was left undisturbed for 10 min. White noise (40 ms) was used as the startle stimulus for all trial types. The startle response was recorded for 400 ms starting with the onset of the startle stimulus. The background noise level was 70 dB. The peak startle amplitude was the dependent variable. A test session consisted of six trial types (e.g., two types of startle-stimulus-only trials, and four types for prepulse inhibition trials). The intensity of the startle stimulus was either 110 or 120 dB. The prepulse sound was presented 100 ms before the onset of the startle stimulus, and its intensity was 74 or 78 dB (20 ms). Four combinations of prepulse and startle stimuli were used (74–110, 78–110, 74–120, and 78–120 dB). Six blocks of the six trial types were presented in a pseudorandom order such that each trial type was presented once within a block. The average inter-trial interval was 15 s (range: 10–20 s). Behavioral tests that were not significantly different between genotypes are listed in Table 2. Behavioral data were obtained automatically by applications (ImageLD, EP, SI, CSI, PS, FZ, TS, HCSI, and BM) based on the public domain Image J program (http://rsb.info.nih.gov/ij/) and modified for each test by the authors. Statistical analysis was conducted using StatView (SAS Institute, Cary, NC). Data were analyzed with the Student’s *t*-test, paired *t*-test, one-way ANOVA, or two-way repeated measures ANOVA. Values in graphs are presented as the mean ± SEM.

## Additional file

Additional file 1: Figure S1. Western blotting analysis of AGR in adult brains of WT and T1KO (*KO*). Brain homogenates prepared from 12-w male mice were subjected to SDS-PAGE and the western blotting was detected using anti-AGR (1:500). Note that the amounts of AGR were not changed in WT and in T1KO. The molecular masses (kDa) are shown in the left. (JPEG 52 kb)
