# Supplementary figures and images for: Abnormalities in perineuronal nets and behavior in mice lacking CSGalNAcT1, a key enzyme in chondroitin sulfate synthesis
Source: Mol Brain. 2017 Oct 5;10:47. doi: 10.1186/s13041-017-0328-5 (PMC5629790; doi:10.1186/s13041-017-0328-5)

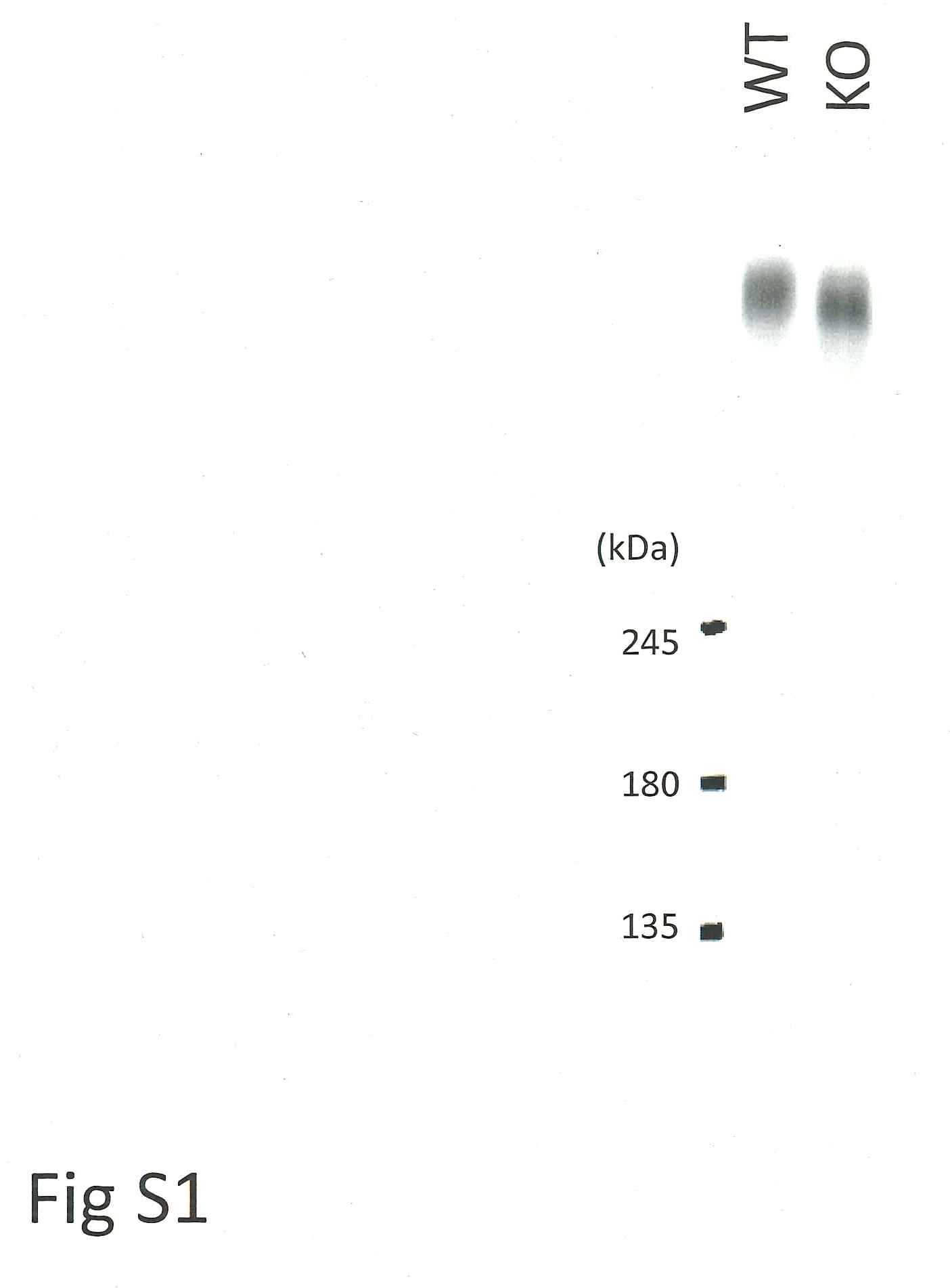

Supplement: Additional file 1: Figure S1. — Western blotting analysis of AGR in adult brains of WT and T1KO (KO). Brain homogenates prepared from 12-w male mice were subjected to SDS-PAGE and the western blotting was detected using anti-AGR (1:500). Note that the amounts of AGR were not changed in WT and in T1KO. The molecular masses (kDa) are shown in the left. (JPEG 52 kb) [file 13041_2017_328_MOESM1_ESM.jpg]
